# Anemia and its associated factors among Adolescents in Kuwait

**DOI:** 10.1038/s41598-020-60816-7

**Published:** 2020-04-03

**Authors:** Lemia Shaban, Abdullah Al-Taiar, Abdur Rahman, Reem Al-Sabah, Olusegun Mojiminiyi

**Affiliations:** 1 Department of Food Science and Nutrition, College of Life Sciences, Kuwait University, Kuwait, Kuwait; 2 School of Community & Environmental Health, College of Health Sciences, Old Dominion University, Norfolk, VA 23529 USA; 3 Department of Community Medicine and Behavioural Sciences, Faculty of Medicine, Kuwait University, Kuwait, Kuwait; 4 Department of Pathology, Faculty of Medicine, Kuwait University, Kuwait, Kuwait

**Keywords:** Epidemiology, Risk factors

## Abstract

We estimated the prevalence of anemia among school children and investigated factors associated with this problem in Kuwait. A cross-sectional study was conducted on 1415 adolescents randomly selected from middle schools in Kuwait. Hemoglobin, iron, ferritin, folate and vitamin B_12_, in addition to many other laboratory indicators, were measured in a venous blood sample. Data on risk factors for anemia were collected from parents and adolescents. Multiple logistic regression was used to investigate factors associated with anemia. The prevalence of anemia was 8.06% (95% CI: 6.69–9.60%), which was significantly higher among females compared to males (10.96% vs. 5.04%; p < 0.001). Mean (SD) Hb level was 133.7 (9.89) g/L and 130.00 (10.48) g/L among males and females, respectively (p < 0.001). The prevalence of mild, moderate and severe anemia was 5.94%, 1.91% and 0.21%, respectively. Gender, age, iron concentration and ferritin were associated with anemia in multivariable analysis. These data indicate that anemia among school children in Kuwait is of mild public health significance. Further reduction in anemia in school girls should focus on correcting iron deficiency. Surveillance systems for anemia may consider using a cut-off point that is specific for the method of blood sampling and the method of Hb measurement.

## Introduction

Anemia is characterized by hemoglobin (Hb) concentration being lower than a specific threshold, and thus creating an impairment in meeting the oxygen demands of tissues^1^. It is a major public health problem with around 1,620 million people worldwide diagnosed with anemia^2^. Generally, a quarter of the world’s population is considered anemic but the prevalence of anemia varies considerably between high-income countries (around 9%) and low-income countries (around 43%)^2^. Due to physiological reasons, pregnant women, women of childbearing age and young children are particularly vulnerable^3^. Anemia has significant implications in terms of mortality^4^, as well as impaired work capacity and economic development^5^. Anemia during childhood has been linked to growth delay, high risk of infections, and poor cognitive and motor development, which may lead to loss of work productivity later in life^3,6^. In fact, anemia is among the top leading causes of disability-adjusted life years lost among adolescents^7^.

Anemia can result from decreased erythrocyte production or increased blood loss, either through hemolysis, bleeding or both. These are determined by nutritional, infectious or genetic factors^8^. Genetic factors are responsible for hemoglobinopathies, such as sickle cell anemia and thalassemia, while in some settings infectious diseases like malaria, soil-transmitted helminths and schistosomiasis are major contributors to anemia. Nutritional anemia results from insufficient nutrients that are needed during Hb synthesis and erythropoiesis. These particularly include iron deficiency (assumed to be responsible for 50% of all anemias^4^), folic acid deficiency, vitamin B_12_ deficiency, vitamin A deficiency and protein-energy malnutrition. In addition, exposure to toxic heavy metals such as lead and low levels of trace elements such as zinc and copper can contribute to anemia^9^. Recent literature also suggests that lower levels of vitamin D can increase the risk of anemia in school children^10^.

Within and between communities, anemia is a sign of socioeconomic disadvantage, with the poorest and least educated being at greatest risk of anemia and its consequences^8,11,12^. In the oil-rich Arab states in the Middle East, like Kuwait, there has been a significant improvement in socioeconomic status in the last few decades. Citizens enjoy a high standard of living that includes free education and medical care, in addition to highly subsidized foods. Furthermore, public health measures regarding proper sewage and sanitation, coupled with environmental and geographical factors, such as arid climate, paucity of vegetation and standing bodies of water, have eliminated the most common parasites that may contribute significantly to anemia. Taken together, one may anticipate that anemia should be a minor health issue in Kuwait and other oil-rich Arab states in the Middle East. Nevertheless, Kuwait Nutritional Surveillance System (KNSS) has consistently reported a prevalence of anemia between 15% and 20% among school children^13^. Furthermore, a previous study reported a prevalence of anemia among school girls to be around 30%^14^, but a fairly recent study reported a prevalence of 8.8% among male and 5.7% among female school children^15^. The later study, however, was not focused on school children and included a sample of people from infancy to adulthood (more than 50 years); and only 182 adolescents between 12–14 years of age were included. The data from KNSS and the above studies caused huge confusion on whether anemia represents a significant health problem in our setting and call for further investigation of the prevalence of anemia in our setting in a systematic manner^13^. In this study, we aimed to estimate the prevalence of anemia among school adolescents and investigate factors associated with anemia in this age group in Kuwait.

## Methods

### Selection and description of participants

This study was a school-based cross-sectional study, conducted in 12 public middle schools selected from all governorates (provinces) in the State of Kuwait. The study population was students between the ages of 11 and 16 years. In each governorate, students were selected using stratified multistage cluster random sampling, with probability proportional to size of schools (i.e. number of students in each school). In this method, a school with a large number of students would have a higher probability of being selected, compared to schools with a small number of students. Blood samples were collected over a three-month period from February through to April 2016. The study was approved by The Ethics Committee at the Ministry of Health, Kuwait (No: 2015/248) as well as The Ethics Committee of the Health Sciences Centre, Kuwait University (No: DR/EC/2338). The study was conducted in accordance with Declaration of Helsinki ethical principles for medical research involving human subjects. A written informed consent from the parents and verbal ascent of each study participant was obtained. Students’ health conditions were recorded in each school; those with major chronic diseases and hemoglobinopathies were excluded from the study and those with self-reported illnesses or minor illnesses were included in the study, however, during data collection this information was noted and was included in the analysis.

### Data collection

Study details have been described previously^16^. In brief, an informed consent form, along with self-administered questionnaire with questions on various sociodemographic variables, were sent to the parents. Upon written consent from parents and verbal assent from the student, a trained interviewer conducted face-to-face interview with the student, using a carefully developed questionnaire that was pilot tested on 20 students who were not included in the study. The questionnaire included structured questions on topics, such as smoking, physical activity, and frequency per week of eating breakfast before going to school. Standing height and body weight of the study subjects were measured in a standardized manner, using digital weight and height scale (Detecto®, Webb City, MO, USA) with the students standing erect without shoes and wearing light clothes.

### Blood collection and biochemical analyses

Upon completion of the interview, students were asked for assent for blood withdrawal (with prior consent of parents). Five ml of venous blood was collected from each student by nurses who are highly trained in obtaining blood from children. Blood analysis was conducted in a major tertiary care hospital, where these tests are routinely performed under strict quality control. Complete blood count (CBC) including Hb, was measured using Beckman Counter Unicel DxH 800 hematology analyzer (Beckman Coulter Inc., Fullerton, CA, USA). As per WHO criteria^17^, anemia was defined among males and females younger than 12 years with Hb less than 115 g/L, while in the age group of 12–14 years with Hb concentration <120 g/L. Anemia was also defined among males 15 years or older who had an Hb concentration <130 g/L and among females 15 years or older with Hb concentration <120 g/L. Hb values were also categorized further into mild, moderate, and severe anemia but this was not used in the analysis, due to the small number of anemic adolescents.

Serum iron, transferrin and ferritin were measured with the Unicel DxI 800 Beckman Coulter analyzer, using commercial kits for serum Fe (Cat. # 467910), ferritin (Cat. # 33020) and transferrin (Cat.# 467942). Percent transferrin saturation was calculated as Fe/[transferrin × 25.6]) ×100. Vitamin B_12_ and folate were analyzed with the Cobas E411 analyzer (Roche Diagnostics GmbH, Mannheim, Germany). Serum vitamin B_12_ was analyzed with the Roche commercial kit (Cat. # 04745736 190), while total folate in hemolyzed whole blood was analyzed with the Roche commercial kits (Cat#s 03253678 122). Red blood cell (RBC) folate was calculated as total folate x 3100/Hct (%), while plasma folate was calculated as total folate –RBC folate. Lead in whole blood (50 µL) was analyzed by ESA’s LeadCare II® Blood Lead Testing System (RNA Medical Bionostics, Inc. Devens, MA, USA) according the manufacturer’s instructions. However, this method was deemed to be not accurate, hence lead was evaluated again in all blood samples using Inductively Coupled Plasma Mass Spectrometry (ICP-MS). As some recent literature suggest that low vitamin D level increase the risk of anemia in children^10^, we measured 25-hydroxyvitamin D using liquid chromatography-tandem mass spectrometry.

### Statistics

Data were double entered into a specifically designed database using Epidata Entry. Data analysis was conducted using Stata (StataCorp2011. Release 12). Body mass index (BMI) was calculated as weight (Kg) divided by height squared (m^2^). Weight status was categorized into normal, overweight and obese according to WHO growth charts. Chi-squared test was used to test differences in categorical variables. Anemia was not common in our setting, hence we used multiple logistic regression to calculate crude and adjusted odds ratios for the association between various factors and anemia. Variables were categorized into sociodemographic factors, nutritional and physical activity habits, in addition to BMI categories, and finally the laboratory factors associated with anemia. Factors that showed association with anemia at less than 20% level of significance were considered in multivariable analysis. Statistical significance of variables was evaluated by the likelihood ratio test, which compares the model with and without the variable. Factors with p < 0.05 were deemed to be statistically significant. Goodness of fit of the final model was evaluated by Hosmer-Lemeshow test. We also used forward and backward selection of variables to identify factors associated with anemia as a confirmatory method. We also used multiple linear regression to investigate the independent factors associated with Hb concentration level and reported this analysis in the text.

## Results

Of 1583 parents approached, 161 refused to participate (child, parents or both). Another 7 samples were not sufficient to conduct blood analysis, thus the analysis below comprised 1415 students. The mean (SD) age was 12.48 (0.94) years and 694 (49.05%) were males. Of 1415 adolescents, 114 were anemic, therefore the prevalence of anemia was 8.06% (95% CI: 6.69–9.60%), which was significantly higher among females compared to males (10.96% vs. 5.04%; p < 0.001). Also, the mean (SD) Hb level was 133.7 (9.89) g/L and 130.00 (10.48) g/L among males and females, respectively (p < 0.001). The prevalence of mild, moderate and severe anemia was 5.94%, 1.91% and 0.21%, respectively. Table 1 shows the mean Hb level, mean corpuscular volume and ferritin concentration by age and gender.

**Table 1:** Mean hemoglobin level, mean corpuscular volume and ferritin concentration by gender and age among 1415 adolescents in Kuwait.

Age (years)^a^	N	Hemoglobin, Mean (SD) g/L	% anemic	Mean Corpuscular Volume (SD) fL	Serum Ferritin, Median (IQR) ng/mL
Total	1415	131.83 (10.36)	8.06	80.56 (6.15)	20.8 (13.9–30.8)
10.3 to <12 year	525	130.45 (9.12)	4.95	79.95 (5.83)	21.7 (14.6–31.2)
12 to <15 year	881	132.58 (10.91)	9.76	80.90 (6.31)	20.4 (13.4–30.4)
Females	721	130.00 (10.48)	10.96	81.04 (6.56)	17.6 (10.7–25.4)
10.3 to <12 year	291	129.89 (9.23)	5.50	80.34 (5.90)	18.7 (11.6–26.5)
12 to <15 year	427	130.14 (11.24)	14.29	81.50 (6.92)	17.1 (9.9–24.3)
Males	694	133.72 (9.89)	5.04	80.06 (5.66)	24.0 (17.9–34.3)
10.3 to <12 year	234	131.16 (8.96)	4.27	79.46 (5.71)	25.7 (19.4–36.6)
12 to <15 year	454	134.88 (10.07)	5.51	80.34 (5.63)	23.4 (17.3–33.7)

^a^Only 9 adolescents (3 females and 6 males) were 15+ years with 2 females being anemic. SD: Standard Deviation IQR: Interquartile Range.

Association between anemia and sociodemographic factors in univariable analysis is shown in Table 2. Of all sociodemographic characteristics, only gender and age of the adolescents were significantly associated with anemia. However, stratified analysis by age group showed that the prevalence of anemia differed significantly only after the age of 12 years (number of cases of anemia was not large enough to investigate the interaction between gender and age in multivariable analysis). With respect to the lifestyle factors, having a meal before going to school, the number of times the child consumed breakfast, lunch or dinner that was not prepared at home all were not associated with anemia (Table 3). Similarly, time spent on physical activity was not associated with anemia, nor was obesity/overweight.

**Table 2:** Association between anemia and socio-demographic factors among 1415 adolescents in univariable analysis.

Characteristics	Total	Prevalence of anemia n (%)	Odds Ratio [95% CI]	p
**Gender**				
Male	694	35 (5.04)	[Ref.]	<0.001
Female	721	79 (10.96)	2.32 [1.53–3.50]	
**Age (year)**				
<12	526	26 (4.94)	[Ref.]	0.003
12-	439	40 (9.11)	1.93 [1.16–3.21]	
≥13	450	48 (10.67)	2.30 [1.40–3.77]	
**Nationality**				
Kuwaiti	1,081	93 (8.60)	[Ref.]	0.174
Non-Kuwait	334	21 (6.29)	0.71 [0.43–1.16]	
Governorate				
**Capital**	155	12 (7.74)	[Ref.]	0.262
Hawally	246	11 (4.47)	56 [0.24–1.30]	
Farawanya	236	21 (8.90)	1.16 [0.55–2.44]	
Jahra	239	20 (8.37)	1.09 [0.51–2.29]	
Mubarak al-Kabeer	148	11 (7.43)	0.95 [0.40–2.24]	
Ahmadi	371	37 (9.97)	1.32 [0.67–2.60]	
**Father’s Education**				
Primary/Intermediate/no formal education	236	23 (9.75)	[Ref.]	0.661
Secondary (high school)	344	24 (6.98)	0.69 [0.38–1.26]	
Diploma	261	21 (8.05)	0.81 [0.46–1.50]	
University & above	541	41 (7.58)	0.76 [0.44–1.30]	
**Mother’s Education**				
Primary/Intermediate/no formal education	183	13 (7.10)	[Ref.]	0.719
Secondary (high school)	304	24 (7.89)	1.12 [0.56–2.26]	
Diploma	303	29 (9.57)	1.38 [0.70–2.74]	
University & above	605	46 (7.60)	1.08 [0.56–2.04]	
**Father’s monthly Income (Kuwaiti Dinar)**				
Less than 500	91	3 (3.30)	[Ref.]	0.477
500 to 1000	304	25 (8.22)	2.63 [0.77–8.91]	
1001 to 1500	421	37 (8.79)	2.83 [0.85–9.38]	
1501 to 2000	219	20 (9.13)	2.95 [0.85–10.18]	
More than 2000	173	12 (6.94)	2.19 [0.60–7.95]	
Do not wish to tell	162	10 (6.17)	1.93 [0.52–7.20]	
**Mother employment**				
Housewife	488	40 (8.20)	[Ref.]	0.285
Paid employment	680	60 (8.82)	1.08 [0.71–1.65]	
Other	219	12 (5.48)	0.64 [0.33–1.26]	
**Mother’s monthly Income (Kuwaiti Dinar)**				
Less than 500	174	8 (4.60)	[Ref.]	0.542
500 to 1000	423	38 (9.00)	2.05 [0.94–4.50]	
1001 to 1500	214	17 (7.94)	1.79 [0.75–4.25]	
1501 to 2000	126	8 (6.35)	1.41 [0.51–3.85]	
More than 2000	36	2 (5.56)	1.22 [0.25–6.00]	
Do not wish to tell	169	14 (8.28)	1.87 [0.76–4.59]	
**Type of housing**				
Rented flat	509	36 (7.07)	[Ref.]	0.259
Rented house	163	9 (5.52)	0.77 [0.36–1.63]	
Owned flat	59	4 (6.78)	0.95 [0.32–2.78]	
Owned house	665	63 (9.47)	1.38 [0.90–2.11]	
**Total number of brother/sisters**				
Zero-two	297	25 (8.42)	[Ref.]	0.787
Three-four	538	40 (7.43)	0.87 [0.51–1.47]	
Five or more	553	47 (8.50)	1.01 [0.60–1.68]

**Table 3:** Association between anemia and nutritional and physical activity habits in addition to body mass index among 1415 adolescents in univariable analysis.

Characteristics	Total	Prevalence of anemia n (%)	Odds Ratio [95%CI]	p
**Times per week consumed breakfast not prepared at home**				
Zero	618	50 (8.09)	[Ref.]	0.913
One-two times	569	49 (8.61)	1.07 [0.71–1.62)	
Three-four	117	8 (6.84)	0.83 [0.38–1.81]	
Five or more	78	6 (7.23)	0.88 [0.36–2.13]	
**Times per week consumed lunch not prepared at home**				
Zero	363	28 (7.71)	[Ref.]	0.158
One-two times	823	65 (7.90)	1.02 [0.64–1.63]	
Three-four	120	16 (13.33)	1.84 [0.95–3.53]	
Five or more	74	4 (5.41)	0.68 [0.23–2.01]	
**Times per week consumed dinner not prepared at home**				
Zero	145	14 (9.66)	[Ref.]	0.904
One-two times	877	71 (8.10)	0.82 [0.45–1.50]	
Three-four	257	21 (8.17)	0.83 [0.41–1.69]	
Five or more	98	7 (7.14)	0.71 [0.28–1.85]	
**Times per week child has breakfast before going to school**				
Every day/Five days a week	569	42 (7.38)	[Ref.]	0.161
Three-four days a week	202	18 (8.91)	1.23 [0.68–2.18]	
One-two days a week	239	13 (5.44)	0.72 [0.38–1.37]	
Never	382	39 (10.21)	1.43 [0.90–2.25]	
**Time spent on physical activity per week**				
Low (lower tertile)	470	42 (8.94)	[Ref.]	0.525
Medium (middle tertile)	474	33 (6.96)	0.76 [0.47–1.23]	
High (higher tertile)	471	39 (8.28)	0.92 [0.58–1.45]	
**Body Mass Index Categories**				
Normal weight	601	44 (7.32)	[Ref.]	0.526
Overweight	320	23 (7.19)	0.98 [0.58–1.65]	
Obese	470	45 (9.57)	1.34 [0.87–2.07]	
Underweight	24	2 (8.33)	1.15 [0.26–5.05]	

In univariable analysis, iron was negatively associated with anemia (p < 0.001), which suggest that anemia is caused by iron deficiency (Table 4). The mean Corpuscular Volume (MCV) was significantly lower among anemic adolescents compared to non-anemic peers, 71.88 (8.74) fL vs. 81.32 (5.23) fL, respectively p < 0.001), which is further evidence of microcytosis due to iron deficiency. Furthermore, low levels of ferritin (less than 15 ng per mL) was significantly associated with anemia (Table 4). Similarly, transferrin saturation showed significant association with anemia. There was no association between the level of lead and anemia in this analysis.

**Table 4:** Association between anemia and laboratory indicators among 1415 adolescents in univariable analysis.

Characteristics	Total	Prevalence of anemia n (%)	Odds Ratio [95% CI]	p
**Iron (µmol/L)**	1412	—	0.87 [0.83–0.90]	<0.001
**Ferritin (ng/mL)**				
≥15 (normal)	1,018	52 (5.11)	[Ref.]	<0.001
<15 (low)	395	62 (15.70)	3.46 [2.34–5.10]	
**Transferrin Saturation (%)**				
<16	617	76 (12.32)	[Ref.]	0.002
≥16	795	38 (4.78)	0.36 [0.24–0.54]	
**Lead (µg/dL)**				
<5	672	47 (6.99)	[Ref.]	0.333
≥5–10	536	46 (8.58)	1.25 [0.82–1.91]	
>10	179	18 (10.06)	1.49 [0.84–2.63]	
**Total Protein (g/L)**	1413	—	1.03 [0.97–1.09]	0.242
<69 (lower tertile)	359	26 (7.24)	[Ref.]	0.375
69 to <72 (middle tertile)	460	33 (7.17)	0.98 [0.58–1.69]	
≥72 (upper tertile)	594	55 (9.26)	1.31 [0.80–2.12]	
**Albumin (g/L)**	1414	—	0.84 [0.77–0.91]	<0.001
≥35	1408	112 (7.95)	[Ref.]	0.078
<35	6	2 (33.33)	5.78 [1.05–31.94]	
**Vitamin B12 (pmol/L)**	1 241	—	1.00 [1.00–1.00]	0.252
<236.7 (lower tertile)	411	40 (9.73)	[Ref.]	0.511
236.7 to <315.45 (middle tertile)	416	35 (8.41)	0.85 [0.53–1.37]	
≥315.45 (upper tertile)	414	31 (7.49)	0.75 [0.46–1.22]	
**RBC Folate (nmol/L)**	1415	—	1.00 [1.00–1.00]	
<1353.5 (lower tertile)	472	40 (9.73)	[Ref.]	0.511
1353.5 to <1596.5 (middle tertile)	416	35 (8.41)	0.85 [0.52–1.37]	
≥1596.5 (upper tertile)	414	31 (7.49)	0.75 [0.45–1.22]	
**Vitamin D status**	1415	—	1.00 [1.00–1.00]	
Severe vitamin D deficiency	558	53 (9.50)	[Ref.]	0.364
Vitamin D deficiency	591	45 (7.61)	0.78 [0.52–1.19]	
Vitamin D insufficiency	215	13 (6.05)	0.61 [0.33–1.15]	
Vitamin D sufficiency	51	3 (5.88)	0.60 [0.18–2.00]	

In multivariable analysis, only gender, age, iron and ferritin levels were associated with anemia (Table 5). Compared to males, females were more likely to be anemic, adjusted odds ratio 1.75 (95% CI: 1.12–2.73), p < 0.001. Similarly, older adolescents were more likely to be anemic compared to those who are younger than 12 years (p=0.014). Iron level was strongly associated with anemia, whether fitted as a continuous or categorical variable, but fitting it as a categorical showed a better fit for the model. Transferrin saturation showed strong collinearity with iron level and had to be removed from the model. Finally, in a supplementary analysis we looked at factors associated with Hb concentration as a continuous outcome. Factors that were significantly associated with Hb concentration in this analysis were gender, age, area of residence, iron, and ferritin. In this analysis, gender, age, iron and ferritin were able to explain 15% of the variability in Hb concentration compared to 21% in the saturated model (i.e. model that includes all variables).

**Table 5:** Factors associated with anemia among 1415 adolescents in multivariable analysis.

Characteristics	Total	Prevalence of anemia n (%)	Odds Ratio [95% CI]	p
**Gender**				
Male	694	35 (5.04)	[Ref.]	<0.014
Female	721	79 (10.96)	1.75 [1.12–2.73]	
**Age (years)**				
<12	526	26 (4.94)	[Ref.]	0.014
12−	439	40 (9.11)	1.98 [1.16–3.35]	
≥13	450	48 (10.67)	2.02 [1.21–3.38]	
**Iron (µmol/L)**				
<9 (first quartile)	301	53 (17.61)	[Ref.]	<0.001
9 to <13 (second quartile)	402	32 (7.96)	0.51 [0.31–0.82]	
13 to <16 (third quartile)	279	12 (4.30)	0.27 [0.14–0.52]	
16+ (fourth quartile)	430	17 (3.95)	0.25 [0.14–0.44]	
**Ferritin (ng/mL)**				
≥15 (normal)	1,018	52 (5.11)	[Ref.]	<0.001
<15 (low)	395	62 (15.70)	2.39 [1.57–3.66]	

## Discussion

There is a paucity of data on the prevalence of anemia in the oil-rich countries in the Middle East, where over the last few decades poverty has been eliminated with a significant improvement in sanitation and access to healthcare services. With the available data, it is not clear whether anemia in school adolescents is a major health problem in Kuwait. On a large nationally representative sample of healthy adolescents, we examined the prevalence of anemia and investigated its underlying risk factors. We found that the prevalence of anemia to be 8% and that anemia was significantly higher among females compared to males. Our findings indicate that anemia in Kuwait is of ‘mild’ public health significance as per WHO classification (prevalence of anemia between 5.0–19.9% of the population)^17^.

We reported a prevalence of anemia in 8% of adolescents, which is lower than that reported from several low-income countries, such as India^18^, Ethiopia^19–21^, Nepal^22^, Brazil^23^, and Latin America^24^. It is similar to that reported from high-income countries like Canada^25^ and The United States of America^26^. In fact, the prevalence of moderate and severe anemia was very low at around 1.91% and 0.21%, respectively. Such low prevalence of anemia in Kuwait is logical and can be explained by several factors. First public health measures, such as proper sewage and sanitation, in addition to the environmental and geographical factors (e.g. arid climate, paucity of vegetation and standing bodies of water), have all eliminated the most common parasites that may contribute significantly to anemia. Second, citizens have a high standard of living, which includes free education and medical care in addition to subsidized foods, such as iron-fortified bread and iron-fortified wheat.

As previously mentioned, KNSS has reported a higher prevalence of anemia among school children, collecting samples for many years on around 13,000 school children per year. For example, the prevalence of anemia among school children was reported to be 14.94%, 17.46% and 19.09% in 2015, 2016 and 2017, respectively^13^. Furthermore, several years ago, a study reported a prevalence of anemia among schoolgirls to be around 30%^14^. These estimates are far higher than our estimate in this study. However, a fairly recent study has estimated the prevalence of anemia in adolescents (12–14 years) to be 8.8% in males and 5.7% in females^15^. Over many years KNSS has used capillary blood that is collected from finger, measuring Hb by the portable HemoCue method. The same method was also used in the earlier study, which estimated the prevalence of anemia in schoolgirls to be 30%^14^. There is a continuous debate on whether measuring Hb from capillary blood underestimates or overestimates the prevalence of anemia compared to venous blood. Some studies have indicated that assessment of Hb concentrations in capillary blood tends to yield higher Hb concentrations compared to venous blood, when samples are analyzed with the same method^27–29^ while other studies reported the opposite findings^30,31^. There is, however, a consensus that there is a significant amount of variability (unreliability) in using capillary blood, which can lead to significant misclassification of anemia status, hence bias in anemia prevalence^29,32^. This issue is complicated further by the continuous debate on the appropriateness of cut-off points used to define anemia and whether they apply universally on all populations^8,33^. A study compared the prevalence of anemia, as per the WHO cut-off point between different countries that have significant differences in the underlying risk factors for anemia and found this cut-off point to be indiscriminating between different populations. As a result, the author suggested to use Hb <90 g/L for moderate-to-severe anemia for anemia surveillance instead of the conventional cut-off point for any anemia^34^. The difference in the estimated prevalence in our study and that of KNSS, or the earlier study that used capillary blood, suggests that a cut-off point in surveillance for anemia should be specific to the blood sampling method (capillary or venous blood), and probably to the method used to assess Hb concentration. It should be clear that these new cut-off points should not be used for medical management of anemia and only used for the purposes of surveillance.

The risk of anemia and its sequelae has been consistently reported to be higher among the poorest and least educated^8,11,12^. In our study, there was no association between anemia and father’s or mother’s education, family income, type of housing or the total number of children (Table 2). This is different from that reported in other low- and middle-income settings, where these factors are strong predictors for anemia^8^. This could be due to the fact that even Kuwaitis with relatively minimum education or wage still have good access to a nutritious diet. Furthermore, some foods, including iron-fortified food items, are highly subsidized.

Females compared to males were more likely to have anemia throughout the analysis. This is reported in many other studies^22,35^ but not all^24^ and is usually explained simply by the loss of iron in menstrual blood loss^3,36^. This is plausible and consistent with our findings that the average ferritin level is significantly higher among males compared to females (Table 1). In fact, 58.86% of female participants have already reached menarche, with ferritin level significantly lower in this group compared to those who did not yet reach menarche (p < 0.001). However, adjusting for ferritin level did not fully explain the higher prevalence of anemia among females compared to males, suggesting that there are unmeasured factors (dietary or other factors) such as higher testosterone in males^37^ that may also contribute to this difference. It is worth noting that we have previously reported higher prevalence of vitamin D deficiency among females compared to males in the same study group^16^. Vitamin D status (as per acceptable cut-off points) was not associated with anemia neither in univariable nor multivariable analysis (Table 4) but categorizing vitamin D in quartiles was associated with anemia in univariable but not multivariable analysis. Also, Hb level showed no association with vitamin D concentration in linear regression analysis (data not shown). This is different from that reported earlier, that poor vitamin D status increases the risk of anemia^10^.

In our setting, the risk of anemia among school children seems to be related to iron deficiency. Factors related to iron deficiency were significant throughout the analysis. This included iron level, ferritin and transferrin saturation, which were all significant predictors for anemia. In fact, these laboratory markers for iron deficiency were significant predictors for Hb concentration in an additional linear regression analysis as well. Furthermore, MCV was significantly lower among anemic adolescents compared to non-anemic adolescents as further evidence that anemia is mainly due to iron deficiency. Vitamin B_12_ was not related to anemia, neither in univariable nor multivariable analysis, while none of the study participants had RBC folate deficiency (<340 nmol/L^38^). When we categorized RBC folate into tertiles, it was not significantly associated with anemia (Table 4). Finally, we have measured lead in all adolescents and found that lead level is not related to anemia in this group of adolescents.

The strength of this study comes from the fact that we studied a large nationally representative sample of adolescents and measured several laboratory markers, including iron and ferritin, in an accredited laboratory. We also collected data on various risk factors from both parents and adolescents. However, we did not investigate infections that may contribute to anemia, such as intestinal parasites. It is worth noting that these infections are unlikely to be a major contributor to anemia in our setting because of the public health measures, such as proper sewage and sanitation in addition to arid climate. Such infections are also usually associated with underweight, which was very rare in our study participants (Table 3).

In conclusion, anemia among school children in Kuwait is of mild public health significance as per WHO classification. Further reduction in anemia in school adolescents in Kuwait may require more focus on female adolescents, in terms of iron deficiency. Our findings showed that previous estimates of anemia prevalence using capillary blood with portable hemoglobinometer (HemoCue) are method-biased and probably far from the true value. Therefore, we suggest that surveillance systems for anemia, including KNSS, should consider using method-based cut-off points that are specific for the type of blood sampling and the method of Hb measurement. This standardization is essential to have a meaningful comparison between different countries and to monitor the trends in the prevalence of anemia over time.

## Data Availability

The datasets generated during and/or analysed during the current study are available from the corresponding author on reasonable request.
